# Sensitive and accurate quantification of human malaria parasites using droplet digital PCR (ddPCR)

**DOI:** 10.1038/srep39183

**Published:** 2016-12-16

**Authors:** Cristian Koepfli, Wang Nguitragool, Natalie E. Hofmann, Leanne J. Robinson, Maria Ome-Kaius, Jetsumon Sattabongkot, Ingrid Felger, Ivo Mueller

**Affiliations:** 1 Walter & Eliza Hall Institute, Parkville, Australia; 2 Department of Medical Biology, University of Melbourne, Parkville, Australia; 3 Faculty of Tropical Medicine, Mahidol University, Bangkok, Thailand; 4 Swiss Tropical and Public Health Institute, Basel, Switzerland; 5 University of Basel, Basel, Switzerland; 6 Papua New Guinea Institute of Medical Research, Madang, Papua New Guinea; 7 Institut Pasteur, Paris, France

## Abstract

Accurate quantification of parasite density in the human host is essential for understanding the biology and pathology of malaria. Semi-quantitative molecular methods are widely applied, but the need for an external standard curve makes it difficult to compare parasite density estimates across studies. Droplet digital PCR (ddPCR) allows direct quantification without the need for a standard curve. ddPCR was used to diagnose and quantify *P. falciparum* and *P. vivax* in clinical patients as well as in asymptomatic samples. ddPCR yielded highly reproducible measurements across the range of parasite densities observed in humans, and showed higher sensitivity than qPCR to diagnose *P. falciparum*, and equal sensitivity for *P. vivax*. Correspondence in quantification was very high (>0.95) between qPCR and ddPCR. Quantification between technical replicates by ddPCR differed 1.5–1.7-fold, compared to 2.4–6.2-fold by qPCR. ddPCR facilitates parasite quantification for studies where absolute densities are required, and will increase comparability of results reported from different laboratories.

The density of malaria parasites in the blood of infected humans ranges from below 1 parasite/μL to tens of thousands of parasites/μL^1^,^2^. The ability of different diagnostic tools to detect infections^3^,^4^, the severity of clinical symptoms^5^,^6^, and transmission potential^7^,^8^ are all closely related to parasite densities. Parasite densities also show pronounced age patterns, reflecting lifetime exposure and naturally acquired immunity on a population level^7^. On a programmatic level, malaria control programs require an understanding of parasite densities and their distribution in the general population to estimate the proportion of infections below the limit of detection of field-deployable diagnostic tools such as light microscopy (LM) or rapid diagnostic tests. This is of particular importance if mass screen and treatment strategies are to be implemented in the field.

For over 100 years densities have been determined by counting parasites by LM. However due to the limited amount of blood examined (normally 0.025–0.0625 μL^9^), parasites are only reliably detected if their density is above 50–100 parasites/μL. Quantification is only possible if densities are above several hundred parasites/μL, e.g. in clinical cases. The increasingly wide-spread use of molecular diagnostic tools has revealed that in most endemic settings 50–80% of all infected individuals carry parasite densities below the limit of detection of microscopy, both for *P. falciparum*^10^ and *P. vivax*^11^. The role of these submicroscopic infections is not yet well understood, in particular their contribution to transmission^12^. Their accurate quantification is essential for epidemiological studies of malaria.

Several quantitative PCR (qPCR) assays have been developed to detect and quantify malaria parasites^13^,^14^,^15^, allowing assessing a much larger volume of blood than LM. Absolute quantification of parasites by qPCR is however challenging: (i) A standard curve must be generated, either from plasmids containing the target sequence of the qPCR, or from cultured ring-stage parasites counted by microscopy. Currently the absence of reference standard curves makes comparison of qPCR results across laboratories difficult. (ii) By microscopy each parasite is counted once, regardless of the stage, but late trophozoites and schizonts contain several genomes. While late *P. falciparum* stages are sequestered in the inner organs^16^, this is expected to be less the case for *P. vivax* and thus a considerable proportion of all circulating parasites might carry several genomes. On the other hand, DNA extraction is rarely fully efficient and a proportion of template is often lost during extraction. As a result, the number of genomes detected may not directly correspond to the number of blood-stage parasites. (iii) PCR efficiency may not be constant across the wide range of template concentration from <1 copy to >10^5^ copies per μL, compromising the precision of quantification especially of very low-density samples^17^.

Droplet digital PCR (ddPCR) is a novel technology that allows absolute quantification of DNA^18^. Each sample is partitioned into approximately 15,000 droplets, which are subject to end-point PCR. The number of droplets with amplification product is then measured, allowing for an estimate of template density without the need for a standard curve. ddPCR has been shown to yield more precise results than qPCR with less variation among technical replicates^19^, and has been successfully applied for the diagnosis and quantification of a number of human pathogens, including vector-borne infections^20^,^21^,^22^.

We assessed the ability of ddPCR to detect and quantify *P. falciparum* and *P. vivax* in both clinical and asymptomatic samples. Results were compared to expert microscopy and an established qPCR targeting the gene encoding 18S ribosomal RNA (rRNA)^14^.

## Results

### Reproducibility and robustness of ddPCR

The samples analyzed in this study are summarized in Table 1. 63 samples positive for *P. falciparum* by qPCR and 53 samples positive for *P. vivax* were run by ddPCR. ddPCR showed solid quantification across 5 orders of magnitude (Supplementary Figure S1). 8 samples per species with densities by ddPCR ranging from <1 copy/μL to 1000 copies/μL were run in triplicate. Reproducibility of results was high, concordance was >0.99 for *P. falciparum* and 0.94–0.99 for *P. vivax*. In 3/4 samples with densities <2 copies/μL by ddPCR 1 or 2 of the triplicates remained negative.

**Figure 1:**
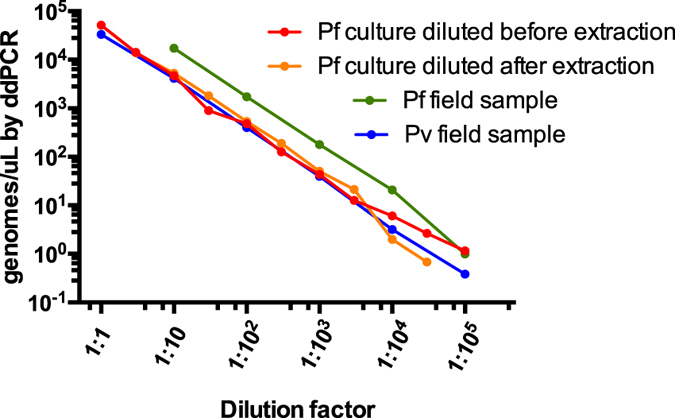
Quantification by ddPCR of a *P. falciparum* culture sample diluted in whole blood before DNA extraction (red), and of DNA from culture (orange) and field samples (green and blue) diluted in H_2_O after extraction.

When extracted DNA from high-density field samples or *P. falciparum* culture was diluted in H_2_O, quantification by ddPCR reflected the dilution steps with very high accuracy (regression slope = 1.001–1.06, *R* > 0.99, Fig. 1). Next, cultured *P. falciparum* was diluted before extraction in uninfected whole blood. Thus the amount of human DNA was kept stable, while the amount of parasite DNA decreased. Across a 10,000-fold dilution, the dilution factor was represented by ddPCR with very high accuracy (regression slope = 0.993, *R* = 0.9962, *P* < 0.001, Fig. 1).

### Absolute quantification by ddPCR, qPCR, and microscopy

**Figure 2:**
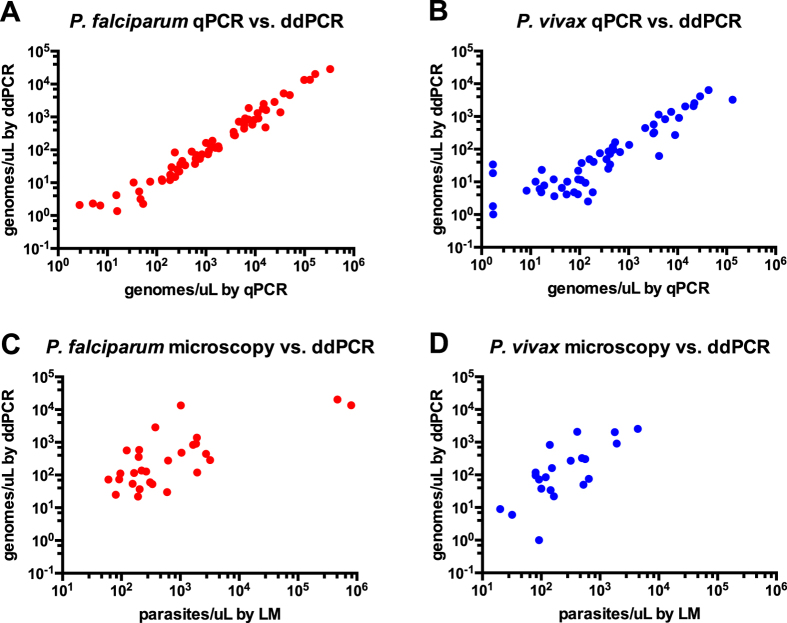
Quantification of parasites by light microscopy, qPCR and ddPCR. Light microscopy data was recorded as parasites/μL, while qPCR and ddPCR data was recorded as DNA copies/μL.

Quantification of field isolates by qPCR (using a circular plasmid as standard) and ddPCR was compared using qPCR positive samples. Samples with densities from 1 copy/μL to 10^6^ copies/μL (by qPCR) were selected from cross-sectional surveys (*Pf*: n = 63, *Pv*: n = 53). Quantification by qPCR and ddPCR was highly correlated for *P. falciparum* (*R* = 0.978, *P* < 0.001, Fig. 2A) and *P. vivax* (*R* = 0.863, *P* < 0.001, Fig. 2B).

Absolute quantification differed significantly between the two methods when a circular plasmid was used to generate a standard curve for qPCR. The quantity estimates by ddPCR were 10.8-fold [CI95: 9.62, 12.06] lower for *P. falciparum* and 5.1-fold [CI95: 3.08, 8.55] lower for *P. vivax* (Table 2). This corresponds to 2–4 cycles by qPCR. Because circular plasmid can result in overestimation of template concentration by qPCR^23^,^24^, plasmids were linearized by restriction digest and qPCR was repeated. Amplification product from linearized plasmids was detected approximately 3 cycles earlier than from supercoiled plasmid of the same concentration (Table 3). When the standard curve generated from linearized plasmids was applied for quantification of samples by qPCR, absolute quantification by qPCR and ddPCR was in a similar range (Table 2), yet remained statistically significant for *P. falciparum* (Student’s T-test: *Pf*: t = −6.44, *P* < 0.001; *Pv*: t = −0.367, *P* = 0.716).

To compare density estimates by microscopy and ddPCR and to assess the number of copies detected per parasite by ddPCR, parasite counts in 0.25 μL blood were done for a subset of the above samples. Correlation between LM and ddPCR was moderate for *P. falciparum* (*R* = 0.727, n = 28, Fig. 2C) and *P. vivax* (*R* = 0.722, n = 21, Fig. 2D) and did not increase when low-density samples (<100 parasites/μL by LM) were excluded. On average 1.41 *P. falciparum* copies/parasite were detected ([CI95 0.32, 2.49]; range 0.02–13.0), and 1.05 *P. vivax* copies/parasite ([CI95 0.35, 1.75]; range 0.008–6.0).

### Sensitivity and accuracy to detect and quantify infections in the general population

In many malaria-endemic regions, in the general population submicroscopic low-density asymptomatic infections predominate. To assess the ability of ddPCR and qPCR to detect such infections, 150 samples collected in the frame of a cross-sectional survey in Papua New Guinea were tested by qPCR and ddPCR. ddPCR and qPCR were each run in triplicate. By microscopy 11 samples were positive for *P. falciparum* and 3 for *P. vivax*.

For *P. falciparum*, 22/150 samples were positive in 3 replicates by ddPCR and qPCR each, and 90 were always negative, with 38 samples positive at least once by ddPCR or qPCR (Table 4). To assess the number of positive samples detected, a sample was counted positive if at least 2/3 replicates yielded a positive result either by ddPCR or by qPCR. ddPCR detected significantly more *P. falciparum* positive samples than qPCR (38 vs. 26, McNemar’s test *P* = 0.006, Table 4).

For *P. vivax*, 10 samples were always positive and 106 samples were always negative (Table 4). Counting samples positive if at least 2/3 replicates were positive for either ddPCR or qPCR, 21 samples were positive by ddPCR and 19 by qPCR, 14 of them by both assays (McNemar’s test *P* = 0.773, Table 4). By qPCR 14 samples were positive in all replicates and 19 in one or two of them. By ddPCR 13 were positive in all replicates and 21 once or twice.

ddPCR was also more sensitive to diagnose mixed infections. Among the 150 samples, 27 were positive in at least one the 3 ddPCR or qPCR replicates for both *P. vivax* and *P. falciparum*. By a single round of ddPCR, 14 of them were diagnosed as mixed infections, but only 6 by qPCR (*P* = 0.024).

Mean densities by ddPCR were 9.91 copies/μL [CI95: 4.83, 20.32] for *P. falciparum* and 2.69 copies/μL [CI95: 1.60, 4.55] for *P. vivax*. Quantification between technical replicates by qPCR differed on average 2.42-fold [CI95: 1.87, 2.97] for *P. falciparum* and 6.16-fold [CI95: 2.20, 10.13] for *P. vivax*. Estimates of densities of technical replicates by ddPCR differed on average 1.51-fold [CI95: 1.32, 1.71] for *P. falciparum* and 1.46-fold [CI95: 1.24, 1.88] for *P. vivax*. Thus variation between replicates by ddPCR was reduced 64.8% for *P. falciparum* and 91.1% for *P. vivax* as compared to qPCR.

## Discussion

ddPCR yielded robust and accurate quantification of *P. falciparum* and *P. vivax* parasites across parasite densities commonly observed in human blood. Importantly, as no standard curve is needed, ddPCR allows for direct comparison of parasite densities measured in different laboratories. Variation in quantification between technical replicates was considerably lower for ddPCR than for qPCR.

Correlation between quantification of qPCR and ddPCR was high, and when linearized plasmid was used for the qPCR standard curve, absolute quantification yielded comparable results. However when circular (supercoiled) plasmid was used, amplification product was detected 2–4 cycles later than corresponding linearized plasmid, and as a consequence densities by qPCR were overestimated as compared to qPCR using the linearized plasmid and to ddPCR. Similar differences when using circular plasmid had been found before, both for *Plasmodium* spp.^15^ and other species^23^. For absolute quantification of parasites by qPCR – and thus to generate values that correspond to LM counts – linearized plasmid must be used.

The assay used targets the 18S rRNA gene, which is a multi-copy gene and 3 copies were amplified with the primers and probes used. Based on expert microscopy counting of a large volume of blood of medium-high density samples, by ddPCR 1–1.4 18S DNA templates per parasite were detected. This suggests that >50% of DNA is lost or sheared during extraction. As a result, copies/μL obtained by ddPCR corresponds well to parasites/μL counted by LM.

The dynamic range of ddPCR coincides well with the densities of infections in field samples. With the protocol applied (i.e. running DNA corresponding to 4 μL whole blood) a sample would be above the dynamic range of ddPCR if above 0.5% of all red blood cells were infected. This is very rarely the case; based on qPCR in a cross-sectional survey in PNG 12/365 *P. falciparum* and none out of 270 *P. vivax* positive samples were above this density^7^. Samples with parasitemia >0.5% can easily be identified, as all droplets in the ddPCR reaction are positive. These samples can subsequently be quantified by diluting the DNA, as quantification by ddPCR represented dilution steps with very high accuracy. In contrast, quantification by qPCR is most reliable if amplification product is detected before cycle 30^17^, yet in several cross-sectional surveys in PNG 52–97% of qPCR-positive samples were detected at cycles >30 (Koepfli, Robinson, Mueller, unpublished). When samples were run in duplicate by qPCR and concentration was below 20 copies/μL, 5–10 fold differences in quantification were frequently observed (Koepfli *et al*. unpublished).

Substantially higher precision in quantification by ddPCR relative to qPCR became also evident when 150 samples collected in a cross-sectional survey in PNG were quantified in triplicate by qPCR and ddPCR. Copy numbers differed on average 2.5–6 fold between duplicates by qPCR when samples were run on different plates, but only 1.5–1.7 fold by ddPCR. This is of particular relevance if densities in the same subject are compared over time, or when genome density is compared to transcript abundance by RT-qPCR to estimate gene expression levels. A 2.5-fold error in each individual qPCR or RT-qPCR reaction can result in significant error in the ratio of parasite densities of paired samples, or in gene expression estimates.

ddPCR detected more *P. falciparum* infections than qPCR in those 150 field samples, while both methods diagnosed a comparable number of *P. vivax* infections. ddPCR also detected more mixed infections. Due to high levels of acquired immunity, most infections were asymptomatic and of low density. This resulted in stochastic amplification; approx. 25% of samples were positive in 1–5 of the total 6 replicates by qPCR and ddPCR, but remained negative in the others. Concentrating DNA during extraction – i.e. eluting the DNA in a smaller volume than the blood volume extracted from – is expected to increase sensitivity of ddPCR and qPCR.

While molecular diagnostic assays are routinely applied in research settings, malaria control programs seldom use them for mass screening or passive case detection. If considering the use of a PCR assay, both laboratory procedures and interpretation of results are similarly easy for ddPCR, qPCR, or conventional PCR and gel electrophoresis. However costs for ddPCR are higher, both for equipment and reagents. A ddPCR set up including droplet generator, PCR machine and droplet reader is available for approx. 100.000 USD, and per sample cost is approx. 5 USD. In comparison qPCR machines are available for 25.000–50.000 USD, and reagents per sample are approx. 2 USD (in both cases DNA extraction at approx. 1.25 USD per sample needs to be added). Pooling several samples per reaction could decrease the costs considerably.

In conclusion, for field studies containing large proportions of low-density infections, quantification of malaria parasites by ddPCR yields more precise results than qPCR, with a similar number of *P. vivax* infections detected by both methods, and a higher number of *P. falciparum* infections detected by ddPCR. Using ddPCR or qPCR with a linearized plasmid as standard, genome densities can be directly compared to parasite densities obtained by microscopy. For studies where absolute quantification is required, reporting ddPCR results will significantly increase comparability of densities reported from different laboratories.

## Materials and Methods

Prior to sample collection, written informed consent was obtained from all study participants, or in case of minors from their parents or legal guardians. The study was approved by PNG IMR IRB, the PNG Medical Research Advisory Committee, the Ethics Committee of the Faculty of Tropical Medicine, Mahidol University, Thailand, and the WEHI Human Research Ethics Committee. All methods were performed in accordance with the relevant guidelines and regulations.

Samples were available from cross-sectional surveys and from clinical cases (Table 1). Cross-sectional surveys were conducted between 2010 and 2014 in Papua New Guinea^7^ (and Koepfli, Robinson, Mueller *et al*. manuscript in preparation) and in Thailand in 2012 (Nguitragool *et al*. submitted). In brief, in each field study 200 μL finger prick blood was collected from 2000–4000 individuals. DNA was extracted using the Favorgen 96-well Genomic DNA Extraction kit and eluted in 200 μL elution buffer. By qPCR *P. falciparum* prevalence was 9–19% in PNG and 1.1% in Thailand, and *P. vivax* prevalence was 13–20% in PNG and 3.3% in Thailand. >50% of infections were submicroscopic, and >90% were asymptomatic.

To assess the correlation in quantification between qPCR and ddPCR across the spectrum of densities observed in human blood, qPCR positive samples with densities ranging from 1 copy/μL to >10^5^ copies/μL were quantified by ddPCR.

To compare estimates of density by microscopy and ddPCR, a subset of the above samples with medium-high densities from the PNG cross-sectionals were selected. Expert microscopists recorded parasites per 2000 white blood cells. This is equivalent to 0.25 μL of whole blood, thus the volume of blood assessed was 10-fold higher than for standard malaria diagnosis^9^.

To compare the ability of qPCR and ddPCR to detect and quantify infections in the general population, 150 samples were selected from a PNG cross-sectional survey irrespective of clinical symptoms or diagnosis by qPCR or LM. In the general population, the large majority of infections are asymptomatic and of low to very-low density^7^. The same samples had also been screened by expert microscopy (parasites per 500 white blood cells). All 150 samples were screened in triplicate by qPCR and ddPCR. These replicates were run on different plates on different days. To generate standard curves for qPCR, a new plasmid dilution series from stock (at a concentration of 10^6^ plasmids/μL) was made for each plate.

A duplex *P. falciparum/P. vivax* ddPCR protocols was used, targeting the 18S ribosomal RNA gene. This gene is present in five copies in the genome, of which three were amplified with the probes and primers used. Primer and probe sequences were derived from published qPCR protocols^14^. The total reaction volume was 22 μL, containing 11 μL BioRad Supermix for Probes (No dUTPs), 4 μL template DNA, and primers and probes in the following concentration: Fal_forward and Fal_reverse 0.16 uM; Fal_Probe 3.2 uM; Viv_forward and Viv_reverse 0.91 uM; Viv_Probe 0.32 uM. Approximately 15000 droplets were generated using the autoDG QX200 droplet generator (BioRad). As in high-density samples some droplets will carry more than one target molecule, the upper limit of the dynamic range is approximately 5-fold the number of droplets (i.e. 75000 targets). The number of targets per droplet follows a Poisson distribution and the total number of targets in the reaction can be calculated based on the proportion of positive droplets^18^. After generating droplets, the following PCR was run: 95° for 10 minutes, 45 cycles of 94° for 30 seconds and 61° for 1 minute, and 98° for 10 minutes, and droplets were counted on a QX200 droplet reader (BioRad).

For comparison of linearized versus supercoiled plasmid as quantification standard in qPCR, TOPO plasmids (Thermo Fisher Scientific) containing the specific target sequence were linearized by EcoRV (New England Biolabs) digest at 37 °C for 1.5 h using 20 U of enzyme in a 50 μL reaction. Linearized and supercoiled plasmids were run in parallel in concentrations of 10^6^, 10^4^ and 10^2^ copies/μL. Assays for *P. falciparum* and *P. vivax* were run in separate tubes using the same 18S rRNA primers and probes^14^, and containing 2 or 4 μL plasmid template in a total volume of 12 μL. A standard curve was generated and the efficiency of the qPCR was calculated. The difference in quantification (Δ*Q*) with linearized vs. supercoiled plasmids was calculated according to equation (1):


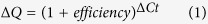


where efficiency was the mean efficiency of the qPCR for linearized and supercoiled plasmid, and Δ*Ct* the difference in cycle number for a given concentration of linearized and supercoiled plasmid. An efficiency of 1 would result in doubling of the amount of DNA in each qPCR cycle. In most qPCR assays, efficiency is below 1.

The assay was run as duplex (VIC for *P. vivax*, 6FAM for *P. falciparum*), and no false-positive droplets for a single channel were observed in control samples extracted from malaria-naïve human volunteers. Samples were called positive if at least 2 droplets were positive for a single channel. In the parasite-free control samples 1–3 droplets positive for both channels (VIC and 6FAM) were detected. Thus samples were only called *P. falciparum/P. vivax* mixed infections by ddPCR if single-positive droplets were observed for both species. Supplementary Figure S2 shows examples of a false positive mixed infection (single positive droplets for *P. vivax* only), and a true mixed infection.

Density values were log10-tranformed to calculate regression between ddPCR, qPCR and microscopy. All copy numbers are reported as geometric mean values.

## Additional Information

**How to cite this article**: Koepfli, C. *et al*. Sensitive and accurate quantification of human malaria parasites using droplet digital PCR (ddPCR). *Sci. Rep.* **6**, 39183; doi: 10.1038/srep39183 (2016).

**Publisher's note:** Springer Nature remains neutral with regard to jurisdictional claims in published maps and institutional affiliations.

## Supplementary Material

Supplementary File S1

Supplementary File S2

## Figures and Tables

**Table 1:** Samples analyzed for this study.

Sample Type	Pf	Pv	Description
*P. falciparum* culture	1		NF54 parasite culture, 4.9% parasitemia, mixed stages. Dilution in uninfected whole blood before extraction, and dilution in H_2_O after extraction
qPCR positive samples	63	53	Samples positive by qPCR, selected from across the range of densities observed in humans. Collected in PNG and Thailand.
LM count samples	28	21	Medium-high density samples, parasites counted by expert microscopy in 0.25 μL blood. These samples are a subset of the qPCR positive samples above.
Cross-sectional samples	150	150	Cross-sectional survey in PNG, including individuals >6 months of age

**Table 2:** Geometric mean copy numbers by qPCR and ddPCR (*Pf*: n = 63, *Pv*: n = 53).

	*P. falciparum* copies/μL [CI95]	*P. vivax* copies/μL [CI95]
qPCR with circular plasmid	2007.3 [1027.3, 3922.3]	174.2 [66.0, 459.4]
qPCR with linear plasmid	267.6 [137.0, 522.9]	37.2 [14.1, 98.4]
ddPCR	162.4 [847, 311.4]	33.9 [16.5, 69.7]

For qPCR either a circular (supercoiled) plasmid or a linearized plasmid was used to generate a standard curve.

**Table 3:** Effect of using linearized vs. supercoiled plasmid for absolute quantification by qPCR.

Plasmid conc.	Linear			Supercoil			ΔCT	Over-estimation
	CT Mean	CT SD	Efficiency	CT Mean	CT SD	Efficiency		
P. falciparum								
1000000	18.96	0.28		22.18	0.09		3.22	7.16
10000	26.68	0.06	0.85	29.81	0.08	0.84	3.13	6.79
100	33.96	0.35		37.29	0.27		3.33	7.67
P. vivax								
1000000	19.34	0.16		22.40	0.10		3.05	6.94
10000	26.76	0.05	0.87	29.34	0.04	0.91	2.58	5.15
100	34.21	0.08		36.68	0.48		2.47	4.78

ΔCT represents the mean difference cycle number between detection of linearized and supercoiled plasmid. The overestimation using supercoiled plasmid was calculated as (1 + efficiency)^ΔCT^. As example, amplification product of *P. falciparum* linearized plasmid at a concentration of 10^6^ copies/μL was detected 3.22 cycles earlier than supercoiled plasmid. The mean efficiency of the reactions was 0.845, thus the difference in estimation of copy numbers was (1.845)^3.^^22^.

**Table 4:** Agreement between qPCR and ddPCR in detection of infections among 150 field samples.

		P. falciparum				P. vivax					
		ddPCR				ddPCR					
		−/−/−	+/−/−	+/+/−	+/+/+	−/−/−	+/−/−	+/+/−	+/+/+		
qPCR	−/−/−	90	16	5	8	106	7	3	1		
	+/−/−	3	1	1	0	7	4	2	1		
	+/+/−	0	1	0	1	3	1	0	1		
	+/+/+	1	0	1	22	0	1	3	10		

qPCR and ddPCR were run in triplicate. Cut off for positivity in ddPCR was set at 2 droplets positive.
