# Supplementary material for: Sensitive and accurate quantification of human malaria parasites using droplet digital PCR (ddPCR)
Source: Sci Rep. 2016 Dec 16;6:39183. doi: 10.1038/srep39183 (PMC5159915; doi:10.1038/srep39183)
Supplement: Supplementary File S1 [file srep39183-s1.pdf]

Sensitive and accurate quantification of human malaria parasites using droplet digital PCR (ddPCR)

Cristian Koepfli, Wang Nguitragool, Natalie E. Hofmann, Leanne J. Robinson, Maria Ome-Kaius, Jetsumon Sattabongkot, Ingrid Felger, Ivo Mueller

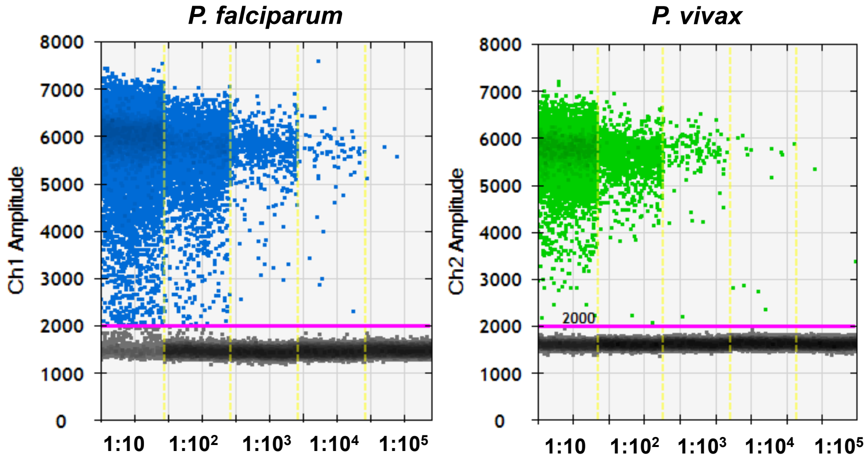

Supplementary Figure S1: Examples of ddPCR runs: Dilution series of high-density *P. falciparum* and *P. vivax* field samples (ranging from 1:10 to $1{:}10^5$ of the original concentration). Positive droplets are indicated in blue (*P. falciparum*) and green (*P. vivax*), negative droplets in gray.

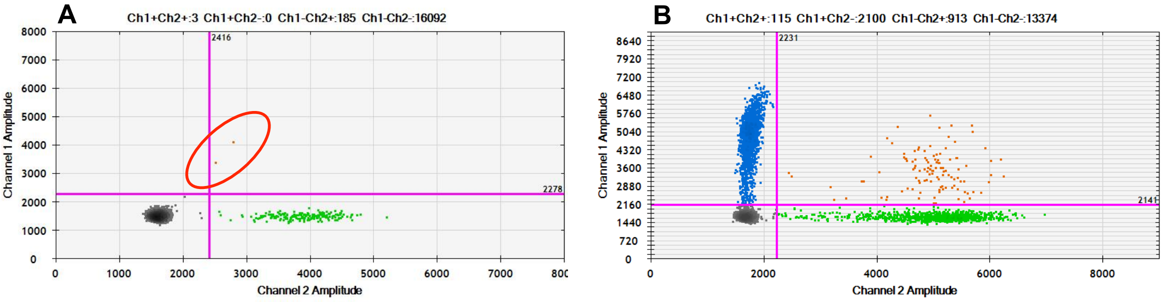

Supplementary Figure S2: Difference between a true *P. falciparum*/*P. vivax* mixed infection and false double-positive droplets. In sample A positive signal is observed for the VIC-channel (*P. vivax*, x-axis), as well as 2 double-positive droplets (red circle). Given that only 185 droplets were positive for VIC, while 16092 were negative for both channels, the chance that these 2 droplets were true *P. falciparum*/*P. vivax* mixed infections is very small. In sample B, single-positive droplets are observed for both channels, as well as a number of droplets positive for both channels. These are true double-positive droplets.
